# Semantic processing of verbal narratives compared to semantic processing of visual narratives: an ERP study of school-aged children

**DOI:** 10.3389/fpsyg.2023.1253509

**Published:** 2024-01-12

**Authors:** Hanna Lindfors, Kristina Hansson, Eric Pakulak, Neil Cohn, Annika Andersson

**Affiliations:** ^1^Linnaeus Language Processing Lab, Department of Swedish, Linnaeus University, Växjö, Sweden; ^2^Logopedics, Phoniatrics and Audiology, Department of Clinical Sciences, Lund University, Lund, Sweden; ^3^Department of Psychology, Stockholm University, Stockholm, Sweden; ^4^Visual Language Lab, Tilburg School of Humanities and Digital Sciences, Department of Communication and Cognition, Tilburg University, Tilburg, Netherlands

**Keywords:** semantic processing, narratives, pictures, comics, children, event related potentials, N400

## Abstract

There is a misconception that pictures are easy to comprehend, which is problematic in pedagogical practices that include pictures. For example, if a child has difficulties with verbal narration to picture sequences, it may be interpreted as specific to spoken language even though the child may have additional difficulties with comprehension of visual narratives in the form of picture sequences. The purpose of the present study was therefore to increase our understanding of semantic processing in the pictorial domain in relation to semantic processing in the verbal domain, focusing on 9–13 years-old children with typical language development. To this end, we measured electrical brain responses (event related potentials, ERPs) in 17 children to (i) pictures (panels) that were predicted versus unpredicted in sequences of panels that conveyed visual narratives and (ii) words that were predicted versus unpredicted in sentences that conveyed verbal narratives. Results demonstrated similarities as there were no significant difference in the magnitude of the N400 effect across domains. The only difference between domains was the predicted difference in distribution, that is, a more posterior N400 effect in the verbal domain than in the pictorial domain. The study contributes to an increased understanding of the complexity of processing of visual narratives and its shared features with processing of verbal narratives, which should be considered in pedagogical practices.

## 1 Introduction

When children’s language proficiency is assessed it is common to use tasks that involve pictures. For instance, a child may be asked to tell a story to sequentially presented pictures and to answer questions about a verbal story told to picture sequences (e.g., Norbury and Bishop, 2003; Gagarina et al., 2012; Boerma et al., 2016). However, this relies on an assumption that picture sequences are unproblematic to comprehend (Coderre, 2020). Yet doubts have been made about this transparency (Cohn, 2020a), leading to consequences for the validity of language assessments. That is, children’s verbal performance should not be interpreted from a language-centered perspective without considering the complexity of visual narratives in the form of picture sequences. If a child struggles to provide a verbal narrative to a visual narrative, it could be due to challenges with verbal language, or with the interpretation of visual narratives *per se*, or with a combination. Indeed, behavioral studies (Zampini et al., 2017; Carlsson et al., 2020; Maryniak, 2022) suggest that children’s comprehension of spoken language might be related to their comprehension of visual narratives (in the articles referred to as nonverbal temporal sequencing, visual story completion, and sequential reasoning). Even so, the relationship between children’s underlying processing in these two domains has yet to be systematically studied. The aim of the present study is therefore to increase our understanding of children’s semantic processing in the pictorial domain in relation to their semantic processing in the verbal domain. By semantic processing, we refer to the use of representations in long-term memory which include, but are not restricted to, language.

Contrary to the assumption that visual narratives are transparent, children need experience to construe the sequences of panels (although single pictures can be iconic) just as they need experience to comprehend spoken language (Cohn, 2020a). Both verbal narratives and visual narratives constitute conventional communication that enable understanding beyond the present. For example, narratives can be conveyed through spoken sentences as well as through wordless comic strips. Further similarities between visual narratives and spoken language have been shown at the cognitive level. Specifically, semantic processing of pictures and words are indexed by the same type of electrophysiological brain response (Kutas and Federmeier, 2011).

However, the similarities in semantic processing of visual narratives and of spoken language have been demonstrated in adults and not in children, with one exception (Manfredi et al., 2020). Unfortunately, Manfredi et al. (2020) did not include a direct statistical comparison between children’s semantic processing in the pictorial and the verbal domain. It is therefore not clear whether there are similarities in children’s semantic processing in the two domains. This is what we target in the present study with a within-subjects design, focusing on children with typical language development.

### 1.1 Semantic processing

The electrophysiological marker of semantic processing referred to as the N400 is an amplitude deflection of a scalp-recorded event-related potential (ERP) with a negative polarity appearing approximately 300–500 ms after stimulus onset, typically broadly distributed and larger over parietal electrode sites. For example, a larger (i.e., more negative) N400 is elicited to the unpredicted word *transmitter* in the sentence “He took a sip from the transmitter” than to the more predictable word *waterfall* in the sentence “He took a sip from the waterfall” (Kutas and Hillyard, 1980). This difference between the amplitude of N400s elicited for experimental conditions is referred to as the N400 effect (Kutas and Federmeier, 2011). Although there are different interpretations of the N400 at the cognitive level, most researchers characterize the N400 as reflecting the degree to which predictions made by the semantic memory system match meaningful stimuli (e.g., words and pictures) presented in experimental designs with varying degrees of predictive context (Kutas and Federmeier, 2011). In line with a general theory of brain activity (Friston and Kiebel, 2009), it has been suggested that the N400 reflects a prediction error within a predictive coding hierarchy (Bornkessel-Schlesewsky and Schlesewsky, 2019; Kuperberg et al., 2020), in which prediction errors with shorter latencies involve less complex stimuli (e.g., tones and phonemes). As such, the N400 can be considered an index of semantic processing as well as an index of predictive processing.

Since the discovery of the N400 to written sentences (Kutas and Hillyard, 1980), it has been demonstrated for different stimulus types (Kutas and Federmeier, 2011) including but not limited to spoken language, visual narratives, music, and math (e.g., Niedeggen and Rösler, 1999; Cohn et al., 2012; Calma-Roddin and Drury, 2020). For instance, a larger N400 is elicited to out-of-key notes compared to correct notes in familiar melodies (Calma-Roddin and Drury, 2020) and to incorrect solutions compared to correct solutions to multiplication problems such as 32 compared to 40 following the presentation of 5 × 8 (Niedeggen and Rösler, 1999). Though the distribution of the N400 effect is similar across modalities (e.g., for written and spoken language; Hagoort and Brown, 2000), it can vary by domain. For example, it is more anterior for prediction errors to pictures than to words (Ganis et al., 1996; Coderre et al., 2018; Manfredi et al., 2020). Consequently, the N400 can be considered to reflect aspects of semantic processing that are both sensitive to domain as well as domain-general (Kutas and Federmeier, 2011).

The N400 is also sensitive to children’s language proficiency, as indicated by age and behavioral measures. A recent review of children younger than 2 years of age (Junge et al., 2021) shows that children with lower language proficiency can have an absent N400 effect or an N400 effect with a longer latency than peers with higher language proficiency. In school-aged children, N400 amplitudes to predicted and unpredicted words in sentences diminish with age until the teenage years (Holcomb et al., 1992). In line with Holcomb et al. (1992), adults have a smaller N400 effect than children aged 5–11 years (Juottonen et al., 1996). Likewise, 7–9 years-olds with a lower word recall ability have larger N400s to both predicted and unpredicted words within sentences compared to 7–9 years-olds with a higher word recall ability (Hampton Wray and Weber-Fox, 2013). Together, these results suggest that smaller N400s and N400 effects reflect more efficient semantic processing and, conversely, that larger N400s and N400 effects reflect a less efficient semantic processing in school-aged children.

In addition to stimuli modality, stimuli domain, and participant characteristics, the N400 has been studied in relation to task demands. The N400 amplitude is similar for passive listening to sentences versus listening combined with tasks, such as judging if a given phoneme is heard in final words of the sentences (Connolly et al., 1990). In these tasks, participants direct their attention to the stimuli, as discussed by Kutas and Federmeier (2011). When participants instead are instructed to ignore certain stimuli and to direct their attention to other stimuli, there is some evidence for a lack of N400 effect for the unattended stimuli (McCarthy and Nobre, 1993). To maintain attention to the stimuli, it is common practice to present auditory stimuli together with unrelated pictures or video clips. However, Weber-Fox et al. (2013) used an ERP paradigm with verbal narratives accompanied by related video clips. This paradigm was adapted for the present study. Because it is more similar to most language processing in naturalistic settings, this paradigm is more ecological valid.

### 1.2 Picture sequences

Picture sequences, such as comic strips that convey narratives and picture manuals that convey instructions, are constructed and comprehended through the use of a visual language according to Visual Language Theory (Cohn, 2013), similar to how a verbal language is used in the construction and comprehension of spoken sentences. Visual languages of comics are systems that involve pictorially presentable meaning consisting of units (i.e., single pictures) and sequences of units with a conventional organization (for details, see Cohn, 2020b).

The target of investigation in the present study is semantic processing of auditorily presented sentences that convey verbal narratives in comparison to semantic processing of picture sequences in the form of wordless comic strips that convey visual narratives. Semantic processing of such visual narratives has been investigated mainly in adults and is indexed by an N400 effect (West and Holcomb, 2002; Cohn et al., 2012; Coderre et al., 2018, 2020). For example, Coderre et al. (2020) presented comic strips without text and the adults demonstrated a fronto-central N400 that was larger for less versus more predictable panels in the comic strips, consistent with how predictability modulates semantic processing of words in sentences.

Semantic processing of visual narratives in children has so far only been investigated in one ERP study (Manfredi et al., 2020). This is unfortunate given the degree to which many language assessments rely on these types of narratives (Coderre, 2020). Manfredi et al. (2020) presented wordless comic strips to typically developing children (*N* = 16, M age = 12.6 years, range = 9–16). Each comic strip consisted of three black and white panels. The final panel in each comic strip either matched or mismatched the content of the comic strip. As anticipated based on results from adult studies of semantic processing of visual narratives, there was a larger fronto-central N400 for unpredicted panels than for predicted panels. The N400 effect occurred together with a P600 effect (measured over parietal sites in the 550–750 ms time window), which was surprising since the ERP effect was restricted to an N400 effect in similar studies with adults (West and Holcomb, 2002; Cohn et al., 2012; Coderre et al., 2018, 2020). However, a biphasic N400/P600 response has been reported in a study where adults were shown short video clips of common human activities (Sitnikova et al., 2003). The participants had an increased fronto-central N400 and an increased posterior positivity (measured in the 600–900 ms time window) to objects that were unpredicted compared to objects that were predicted in the activities (e.g., ironing with a knife or an iron). This inconsistent occurrence of P600 effects after N400 effects in the pictorial domain parallels that of the verbal domain (Münte et al., 1998; Faustmann et al., 2005; for a review, see Van Petten and Luka, 2012).

Another similarity between semantic processing in the pictorial domain and the verbal domain is a modulation by proficiency. N400s for visual narratives in adults are modulated by both the frequency of reading visual narratives and the age at which people began reading comics (a proxy for “age of acquisition”) as measured with the questionnaire called Visual Language Fluency Index (Coderre and Cohn, 2023). Likewise, larger N400s in the verbal domain are related to lower language ability in school-aged children, as indexed by language disorder, lower word recall performance and lower age (see review in the previous section).

Returning to Manfredi et al. (2020), in addition to study children’s semantic processing of visual narratives, Manfredi et al. investigated semantic processing of sentences and compared the ERP effects in typically developing children with ERP effects in children with autism spectrum disorder (no significant group difference in general intelligence measured with WISC-III). Both groups had an N400 effect (but no P600 effect) of semantic prediction to final words in isolated three-word sentences (subject, verb, and object, e.g., *Paulo eats pasta/poem*). This result for sentences differed from the result for the visual narratives, in which the children with autism spectrum disorder had an isolated N400 effect while the children with typical development had a biphasic N400/P600 response. The P600 effect in children with typical development could indicate that they, in contrast to the children with autism spectrum disorder, recognized the discontinuity of the visual information relative to its prior context and therefore reanalysed the presented stimuli, according to Manfredi et al. (2020).

The Manfredi et al. (2020) study did not explore associations between semantic processing of sentences and visual narratives, but comparisons between children’s behavioral performances in these domains have been the focus of a few studies. In a behavioral study of children’s comprehension of narratives, children answered questions on the content equally well when the narrative was presented verbally as when it was presented as picture sequences (Bishop and Adams, 1992). Yet, it is difficult to draw conclusions about the children’s ability in the pictorial domain since the measures were based on their verbal performance. Indeed, visual and verbal abilities are often conflated in behavioral studies of children’s development of visual narrative comprehension, as noted in a recent review (Cohn, 2020b).

There is, however, a type of behavioral test of visual narrative comprehension that does not rely on children giving verbal answers. In this type of test, children are required either to select a picture that completes a picture sequence that conveys a visual narrative, or to arrange several pictures in a sequence that conveys a visual narrative. Integrative analysis of results of multiple studies of picture arrangement tasks show that children improve in a steady by-age development, and these and other results suggest sequential continuity is only understood between 4 and 6 years of age, with performance improving until the early teens (Cohn, 2020a). In a study of the association between children’s performance on a picture arrangement test (called sequential reasoning) and their performance on a standardized receptive language test, Zampini et al. (2017) reported a positive correlation, and this association has been replicated in other similar behavioral studies on children (Carlsson et al., 2020; Maryniak, 2022). Together, these studies suggest an association between children’s verbal comprehension and visual narrative comprehension. However, it is important to note that these studies did not consider participants’ familiarity with visual narratives.

Even though picture sequence completion and picture arrangement tests do not rely on children’s verbal performance, they still require an overt response from children (i.e., picture selection or picture arrangement). An overt response from children is also required in receptive language tests, for example selecting pictures that match auditorily presented sentences. It is therefore conceivable that children’s motivation and general ability to perform on such tests could be confounding factors in behavioral studies of relationships between children’s comprehension in the verbal and the pictorial domain. Another confounding factor could arise if children use language to solve tasks in the pictorial domain even if the tasks do not require verbal responses.

### 1.3 Present study

This study aims to increase our understanding of semantic processing in the pictorial domain in relation to semantic processing in the verbal domain in children with typical language development, since there are few studies on children. To this end, we measured ERPs to panels that were predicted versus unpredicted in the context of picture sequences that conveyed wordless narratives (i.e., visual narratives), and to words that were predicted versus unpredicted in the context of sentences that conveyed verbal narratives. This methodology has several advantages. Measurements of ERPs can reveal the earliest stages of children’s pictorial and verbal processing independent of their ability to perform on tests requiring overt answers. The use of narratives provides a context that increases ecological validity since everyday communication occurs in rich contexts. Indeed, there is a need to study language processing in contexts beyond isolated sentences (Hasson et al., 2018). Crucially, the present study design permits a direct, within-subjects, analysis of semantic processing in both the pictorial and the verbal domain.

In line with previous research, as reviewed above, we expected an N400 effect of semantic prediction both for picture narratives and verbal narratives, with a frontal maximum and a parietal maximum respectively, suggesting similar processing of meaning across domains. The results will have implications for our understanding of semantic processing in children with typical language development and will provide a foundation for subsequent investigations of semantic processing in children with language disorder. This will in turn inform the development and refinement of language assessments and interventions.

## 2 Method

### 2.1 Participants

In total, 18 children were recruited, of which one participant was excluded due to a technical error which prevented stimuli presentation. Consequently, the final sample consisted of 17 participants. Children aged 9–13 years were recruited as we expected them to be able to attend to both the pictorial and the verbal paradigm. Inclusion criteria were right-handedness, Swedish as a first language, normal vision (or corrected to normal), normal hearing, and lack of neurodevelopmental disorders (e.g., autism spectrum disorder, attention deficit disorder, and developmental language disorder). To characterize the sample, background measures involved socioeconomic status, non-verbal cognitive ability, verbal proficiency, and visual narrative proficiency.

The measure of socioeconomic status (SES) was obtained with a questionnaire regarding caregivers’ educational level, in which a minimum score of one represents less than 7 years in school and a maximum score of seven represents an advanced graduate degree (Hollingshead, 1975).

The standardized tests Recalling Sentences from the Swedish version of Clinical Evaluation of Language Fundamentals (CELF-4, Semel et al., 2013) and the digital short version of Raven’s 2 Progressive Matrices Clinical Edition (Raven’s 2, Raven et al., 2018) were used to confirm typical verbal and non-verbal cognitive abilities. To reduce the duration of the behavioral testing, we included only one out of the four tests from CELF-4 that according to the manual are used to calculate a composite score measuring general language ability. Recalling Sentences is a sentence repetition task which can be considered to measure language ability broadly, engaging representations of meaning and form at many linguistic levels (e.g., phonological, morphological, syntactic) and loading on an underlying language ability construct (Klem et al., 2015; Moll et al., 2015). Sentence repetition has been shown to be a sensitivite and specific measure of language proficiency (Conti-Ramsden et al., 2001; Thordardottir and Brandeker, 2013; Taha et al., 2021). This has not yet been evaluated for Swedish, but the measure has Scandinavian norms (*N* = 600) and shows high reliability (*r_xx_* = 0.89) (Semel et al., 2013). The digital short version of Raven’s 2 also has good psychometric properties (Raven et al., 2018). Raw scores for Recalling Sentences from CELF-4 and raw scores for Raven’s 2 were transformed to percentiles. For both tests scores below the 16th percentile are considered below average, and above the 84th percentile above average.

Visual narrative proficiency was measured with the Visual Language Fluency Index (VLFI) questionnaire (Cohn, 2014, 2020a) which was translated into Swedish and adapted to children (see Supplementary File). The original questionnaire asks participants to rate their reading and drawing habits for comics, both currently and during childhood, but adaptation for children excluded the questions for childhood habits. In calculating VLFI scores, excluded childhood questions were given the same raw scores as responses to questions about current use, which would not affect the derivation of calculated VLFI scores (Cohn, 2014). A VLFI score below eight indexes low fluency, 12–19 average fluency and above 20 high fluency, and this range has been corroborated by analysis of almost 2,000 VLFI surveys across ages of adults (Cohn, 2020a). However, the VLFI has not been validated for children, and the present results should therefore be interpreted accordingly.

**Figure 1:**
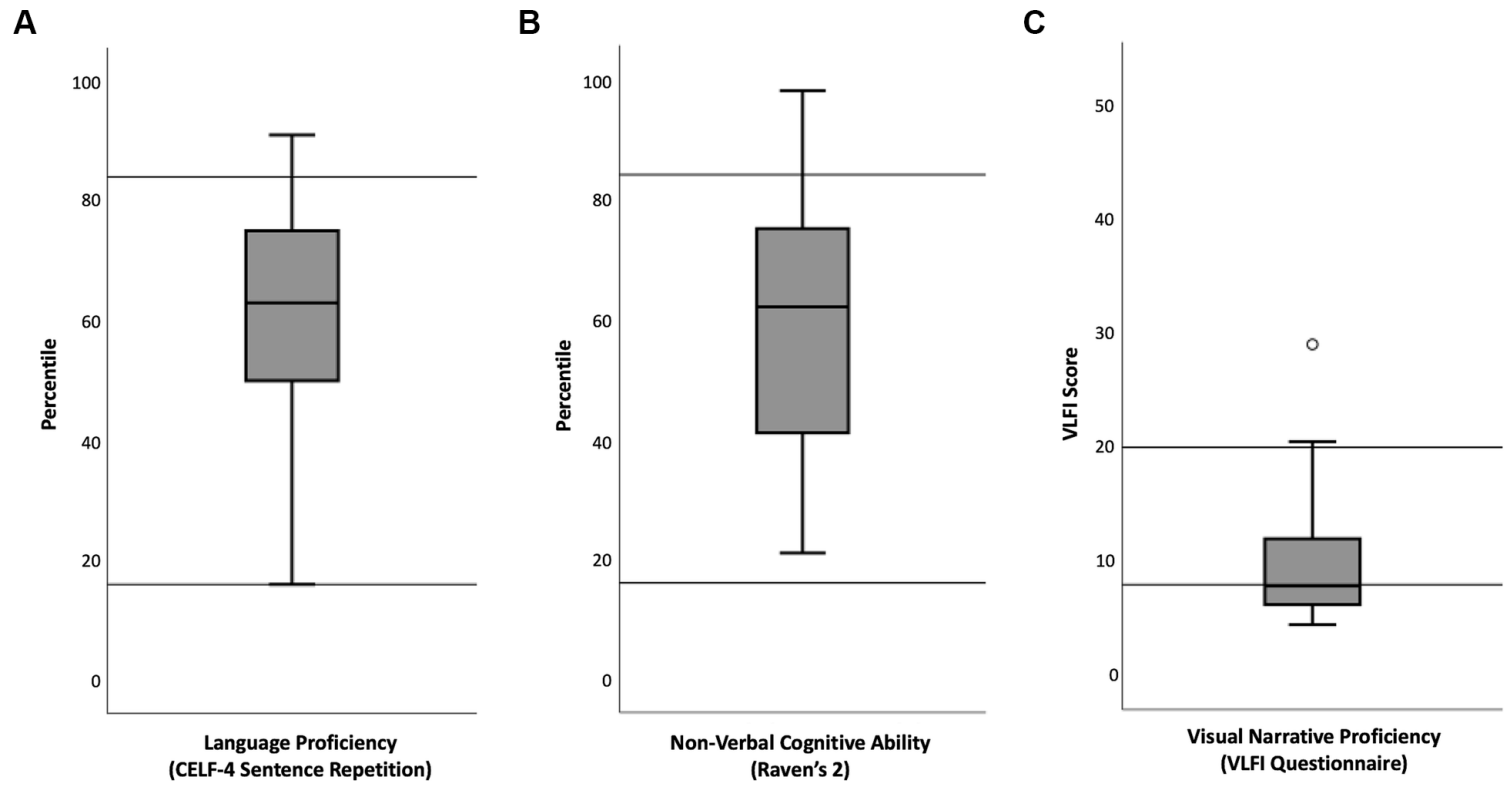
Boxplots of participants’ scores on standardized verbal and non-verbal behavioral tests. Horizontal lines in **(A)** and **(B)** mark the 16th percentile and the 84th percentile, which are the thresholds for below and above average scores. Horizontal lines in **(C)** mark 8 VLFI scores and 20 VLFI scores, which are thresholds for low and high scores in adults (VLFI is not standardized for children). The maximum possible VLFI score is 52.5.

Participant characteristics are summarized in Table 1 and Figure 1. There were no statistically significant differences between girls (*N* = 13) and boys (*N* = 4) on any of the measures (all *p*s > 0.35). Caregivers’ educational level (averaged for the caregivers of each child) ranged from upper secondary education (12 years of schooling) to advanced graduate degree. Scores on the Recalling Sentences task (M = 57th percentile, SD = 24) and Raven’s 2 (M = 59th percentile, SD = 23) confirmed that the children had typical verbal and non-verbal cognitive abilities. One child did not answer the VLFI questionnaire. The mean VLFI score for the remaining 16 children was 10.6 (SD = 6.8, range 4.5–29) which would indicate a low to average visual narrative proficiency in adult scales. Raw scores on VLFI questions regarding comics reading frequency (question 4–7, see Supplementary material) suggest that most of the participating children never or almost never read comic books (75%), comic strips (81%), graphic novels (50%), or Manga (94%).

**Table 1:** Participant characteristics.

	Age	SES	Comics reading frequency			
			Comic books	Comic strips	Graphic novels	Manga
M (SD) Range	11;3 (1;0) 9;4–13;1	5.6 (0.7) 4–7	2.1 (1.7) 1–7	2.1 (1.7) 1–7	2.6 (1.7) 1–7	1.2 (0.5) 1–3

Age in years; months. Caregivers’ education as proxy for socioeconomic status (SES) with a maximum score of seven. Raw scores on question 4–7 of VLFI (see Supplementary material) indicating comics reading frequency (1 = never, 2 = almost never, 3 = sometimes, 4 = quite often, 5 = often, 6 = almost always, 7 = always).

### 2.2 Verbal stimuli

**Figure 2:**
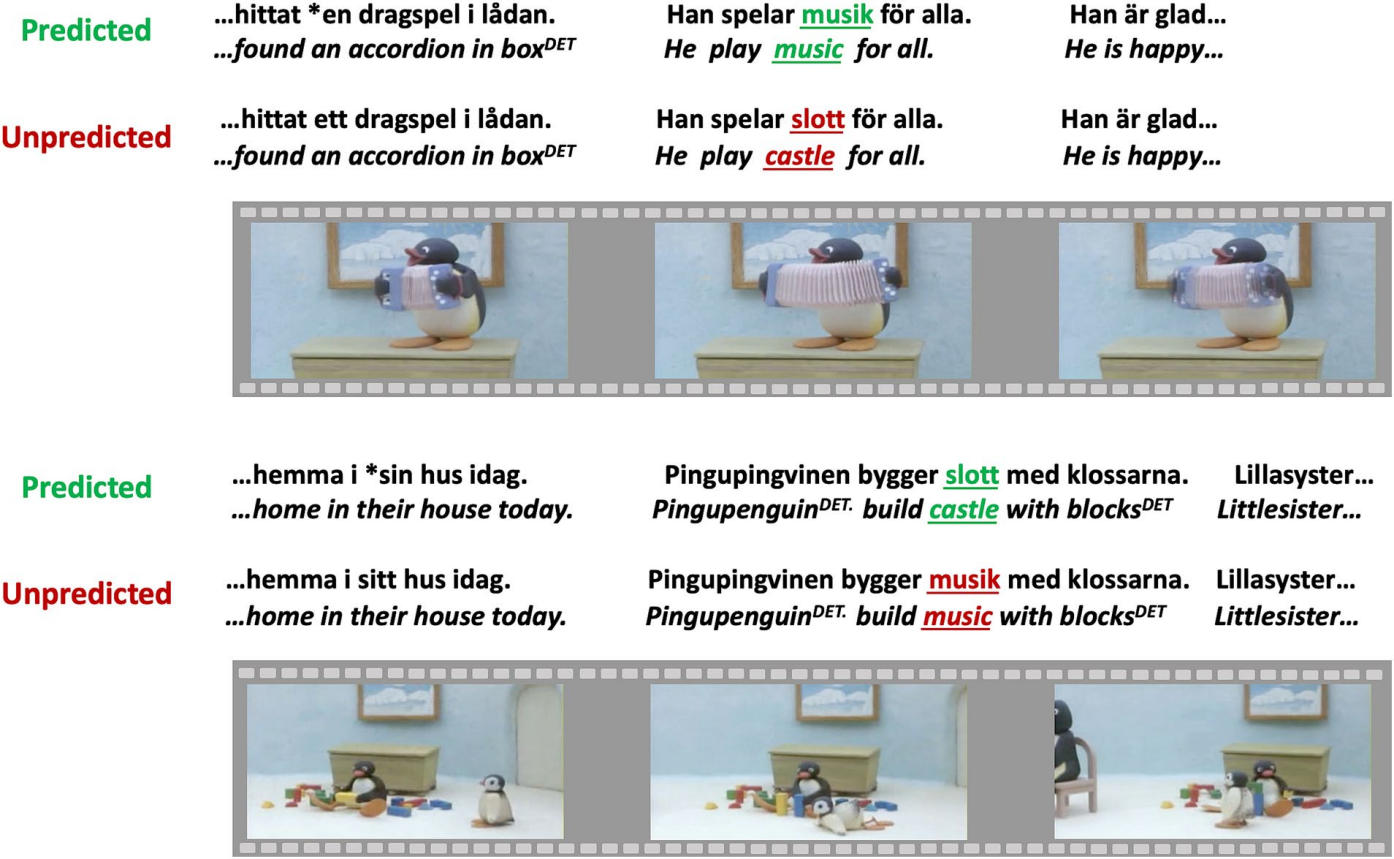
Examples of verbal stimuli. Critical words are underlined and green when predicted while the same words are in red when unpredicted from the context. * Marks a violation of gender congruence that is considered a filler for the purposes of the current analyses of semantic processing. Swedish stimulus sentences with literal translations into English underneath in italics. ^DET^ indicates a post-nominal determiner. Images sourced from https://archive.org/details/Pingu_Season_1to6/aaf-pingu.s01e17.pingu.has.music.lessons.dvdrip.xvid.avi. Reproduced under Public Domain Mark 1.0, https://creativecommons.org/publicdomain/mark/1.0/.

Verbal narratives were developed to accompany ten claymation videos featuring the penguin character Pingu and his family, produced by Sony Music Entertainment Inc. These ten verbal narratives contained 100 Swedish sentences each. Critical words had medial positions in sentences and were concrete nouns considered by the authors to be acquired before the age of 9 years. Ten sentences per verbal narrative contained a predicted critical word and ten sentences per verbal narrative contained an unpredicted critical word (Figure 2). In other words, 10% of the sentences in each verbal narrative contained an unpredicted critical word. Control versions of the ten verbal narratives were developed where predicted words in one version appeared as unpredicted words in the other, and vice versa. This ensured that specific words were unrelated to any ERP effects. Of the remaining 80 sentences per verbal narrative, 30 sentences were fillers and 50 sentences contained grammatical manipulations that are not part of the present study. The order of sentences was pseudorandomized for each claymation video so that there were no more than three consecutive sentences of the same type and no more than three consecutive sentences with linguistic violations.

The sentences were recorded with a professional actress. Thereafter, the claymation video was modified in length by an experienced movie editor to correspond to the length of the recorded verbal narratives; this was necessary as in some cases the experimental manipulations made the verbal narratives longer than the original claymation video. The timing between all sentence onsets and the frames of the claymation videos was identical for the two versions of each verbal narrative that accompanied the same claymation video. The distinction between an unpredictable critical word and a predictable critical word in each respective version was therefore specific to the verbal domain.

Each participant was presented with five different claymation videos, with a duration of approximately 7 min per video. Each video was started by a keypress and was initiated with a fixation cross. Participants were instructed to watch the claymation videos and to listen to the verbal narratives for comprehension. They were informed that some words would not fit the narrative context.

### 2.3 Picture stimuli

**Figure 3:**
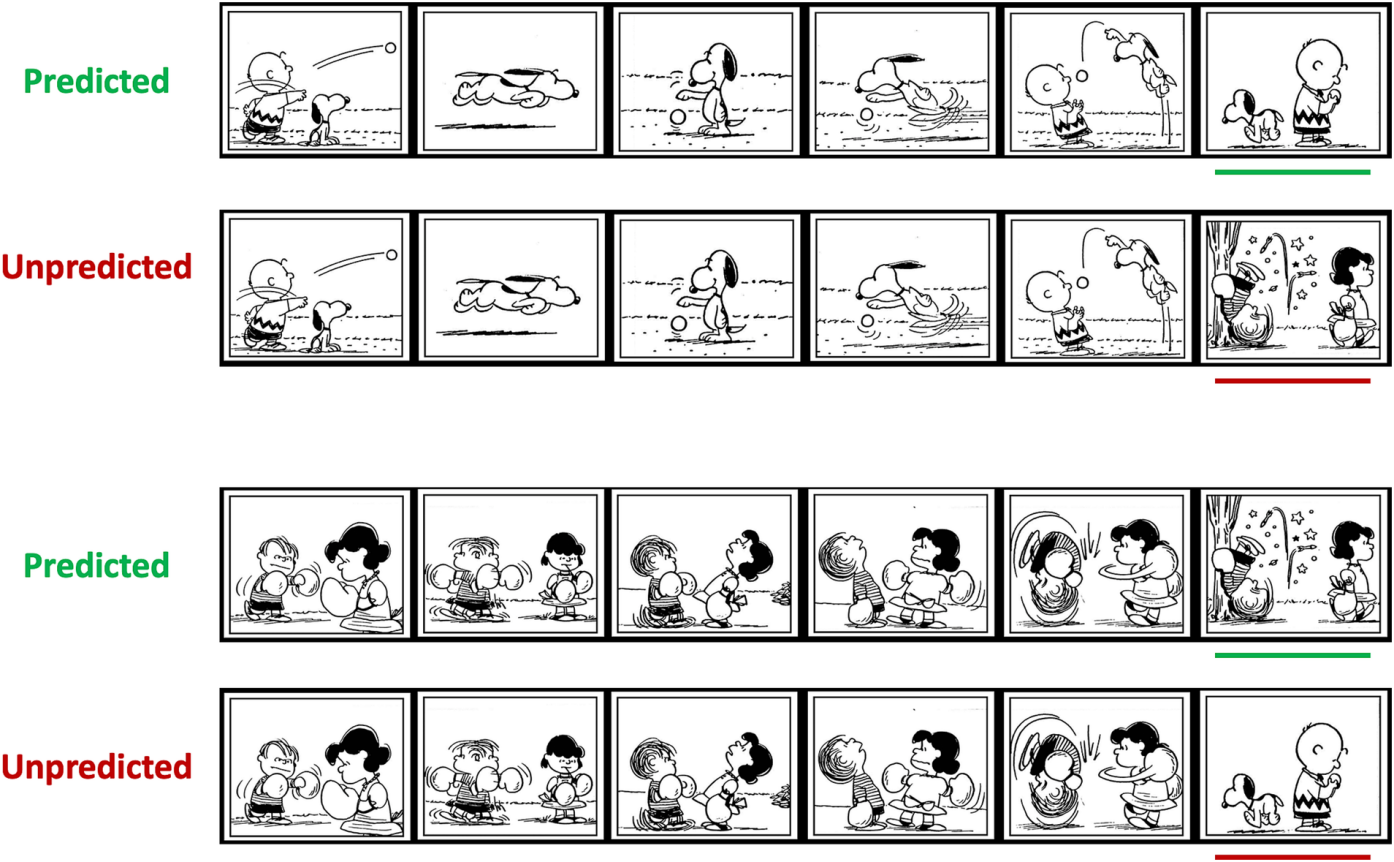
Examples of picture stimuli. Each comic strip consisted of six panels that conveyed a visual narrative. Panels were presented one at a time. Green lines mark predictable critical panels and red lines mark unpredictable critical panels (for visualization here only). Images are copyright Peanuts Worldwide. Reproduced with permission.

Similar to the verbal stimuli, picture stimuli contained manipulations of prediction within narrative contexts. The panels, which have been used previously (Cohn et al., 2014), consisted of 3 sets with 210 comic strips (*Peanuts* made by Charles M. Schulz) per set. Each strip contained six black and white panels that conveyed a visual narrative (Figure 3). Each set had 45 comic strips that ended with a predicted panel and 45 comic strips that ended with an unpredicted panel. There were control versions of the sets where predicted panels in initial versions appeared as unpredicted panels, and vice versa. This ensured that specific panels were unrelated to any ERP effects. The remaining 120 comic strips per set contained manipulations that are not part of the present study.

Each comic strip was initiated with a fixation cross with a duration of 500 ms, followed by a 500 ms ISI before the presentation of the first panel. Panels were presented with a duration of 1,350 ms per panel and a 300 ms ISI between panels, consistent with the Cohn et al. (2014) study. The final panel of each comic strip was followed by a question mark to prompt a key response, after which the next comic strip started. The keys were on a hand-held pad and participants held one thumb on a green key and one thumb on a red key. They were instructed to press the green key when a comic strip was easy to comprehend and the red key when a comic strip was difficult to comprehend, to replicate the task used in the only previous ERP study of children’s visual narrative processing (Manfredi et al., 2020). Response hands were counterbalanced across participants and sets. Participants were informed that some panels would not match the narrative context.

### 2.4 Procedure

Children and their caregivers provided oral and written informed consent (approved by the Swedish Ethical Review Authority) prior to their participation. Children filled out two questionnaires together with their caregivers, one to confirm righthandedness (Oldfield, 1971) and one regarding their visual narrative proficiency (VLFI). In addition, caregivers filled out a questionnaire concerning their educational level (Hollingshead, 1975). The subsequent ERP recordings consisted of two parts. The picture stimuli were presented first and had a duration of about 50 min. The verbal stimuli were then presented and had a duration of about 35 min. Stimuli were presented with PsychoPy (Peirce et al., 2019). Children were instructed to take a pause halfway through the presentation of the picture stimuli to stay alert and to take additional pauses when needed. After the ERP recording, the two standardized behavioral tests were administered by one of the authors with extensive experience in testing children, which took approximately 20 min in total. Children received a university hoodie for their participation.

### 2.5 EEG recordings and processing

EEG was recorded with BrainProducts ActiCap with 32 active electrodes, with the left mastoid as an online reference and with Fpz as ground. Electrodes were also placed at the outer canthi of each eye and at the forehead, for recordings of ocular artifacts together with Fp1/2. The impedance was kept below 25 kΩ. Sampling rate was set at 250 Hz.

The EEG was processed offline with EEGLAB (Delorme and Makeig, 2004) in MATLAB (The MathWorks Inc., 2020). First, the EEG was filtered with a 0.1–100 Hz bandpass filter with the EEGLAB default transition band. We manually rejected EEG segments with excessive muscle artifacts. Thereafter, we applied a 30 Hz low pass filter with the EEGLAB default transition band. The EEG was then ICA decomposed and ICA components corresponding to blinks and horizontal eye movements were identified and subsequently rejected. Next, the EEG was re-referenced to the averaged mastoids. Relative to the onsets of critical pictures and words, epochs were extracted with a 1,200 ms duration with a −200 ms baseline correction.

### 2.6 Analyses

**Figure 4:**
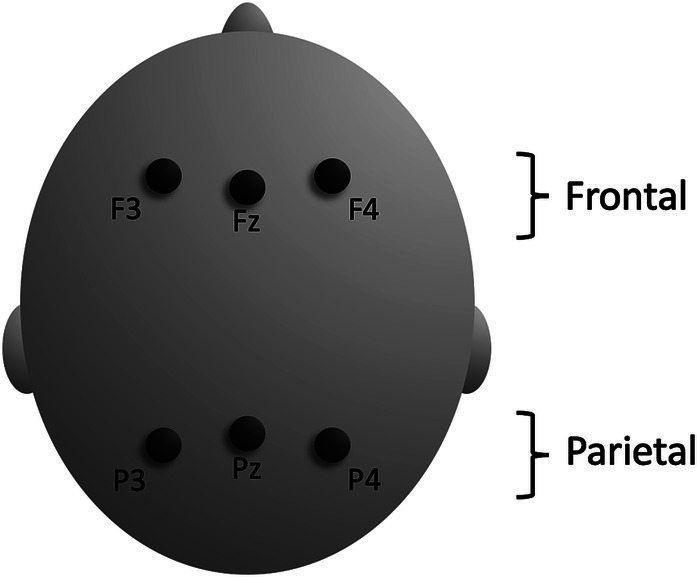
Schematic illustration of scalp electrodes used for ERP analyses.

The ERP measures were obtained by averaging mean amplitudes over frontal and parietal electrode sites (F3, Fz, F4, P3, Pz, P4) in two time-windows (300–500 ms, 500–700 ms) of the epoch for each condition (predicted/unpredicted), each domain (pictorial/verbal), and for each participant. The 300–500 ms time window was determined in accordance with recommendations in a review of methodology in N400 studies (Šoškić et al., 2022). The 500–700 ms time window was included as children can have prolonged N400 effects compared to adults and to allow for an exploration of the inconsistent occurrence of P600 effects following N400 effects. The frontal and parietal electrode sites (Figure 4) were selected to include sites where the N400 effect is typically maximal in each domain. This selection was based on previous research and not on the present data to reduce the risk of a spurious finding (Luck and Gaspelin, 2017). There were no significant differences between the number of included trials per domain (verbal/visual) or condition (predicted/unpredicted) (Table 2), domain *F*(1, 16) = 3.10, *p* = 0.097, *η_p_*^2^ = 0.16, condition *F*(1, 16) = 2.73, *p* = 0.118, *η_p_*^2^ = 0.15, domain by condition *F* < 1.

**Table 2:** Included trials per condition.

	Verbal paradigm		Visual paradigm	
	Predicted word	Unpredicted word	Predicted panel	Unpredicted panel
M (SE) Range	43.8 (1.6) 29–50	44.9 (1.5) 27–50	40.9 (1.3) 25–45	41.5 (1.2) 29–45

N400 effects in each domain were calculated by subtracting the ERP measures for the predicted condition from the ERP measures for the unpredicted condition. To investigate the magnitudes of N400 effects in the pictorial and the verbal domain, and the hypothesized distribution difference between the domains, a repeated measures ANOVA was computed. This analysis included the factor *domain* (pictorial/verbal) and the interaction domain^*^distribution (frontal/parietal) for each time window. Marginally significant interactions (*p* < 0.1) were explored with pairwise comparisons for the frontal and parietal electrode sites separately, with Bonferroni correction to correct for multiple comparisons. Waveforms were plotted with ERPLAB (Lopez-Calderon and Luck, 2014) in MATLAB (The MathWorks Inc., 2020).

**Figure 5:**
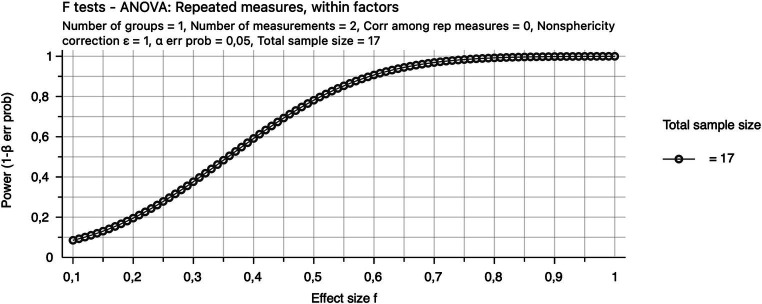
Sensitivity power plot.

Pre-study power calculations were not performed, but the sample size is comparable to the mean sample size (18.5 participants) in recent N400 studies reviewed by Šoškić et al. (2022). In addition, we provide a sensitivity power plot (created in G*Power: Faul et al., 2007). A sensitivity power plot facilitates the interpretation of results when pre-study power calculations have not been performed (Lakens, 2022). The sensitivity power plot below (Figure 5) shows that a within-subjects ANOVA with two measurements and 17 participants has reasonable power for the detection of differences with large effect sizes (e.g., 0.8 for *f* = 0.5).

## 3 Results

### 3.1 ERP responses to verbal narratives

**Figure 6:**
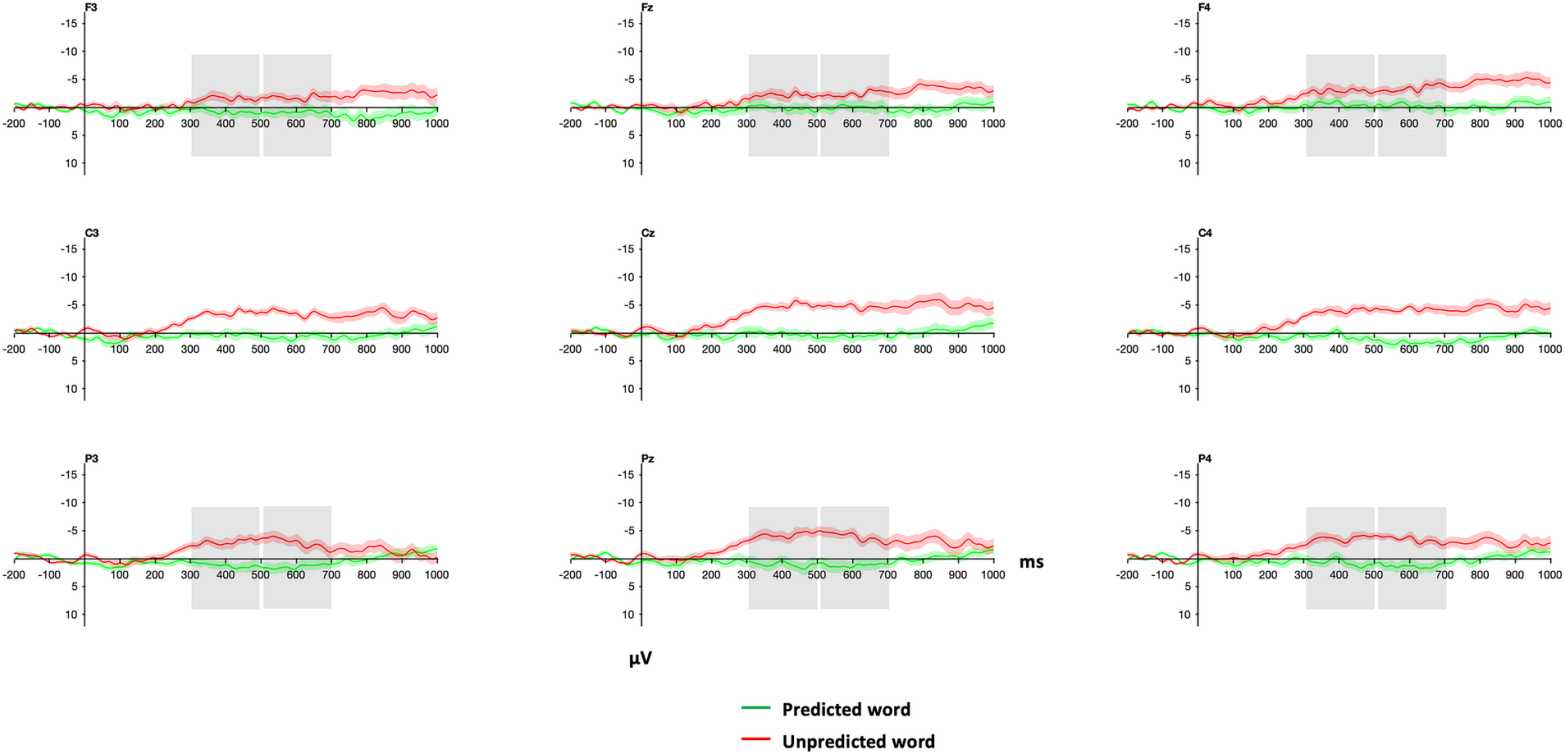
Grand average waveforms of ERPs for predicted and unpredicted words within verbal narratives. Word onsets of critical words are at zero ms. Grey boxes indicate the time windows and electrodes that were used in the statistical analyses (see 3.3 below). Red and green shadings indicate ±1 SE. Negative is plotted up.

Visual inspection of Figure 6 shows that the auditorily presented words elicited a more negative ERP response when unpredicted from the context than when predicted in the 300–500 ms and 500–700 ms time window.

### 3.2 ERP responses to visual narratives

**Figure 7:**
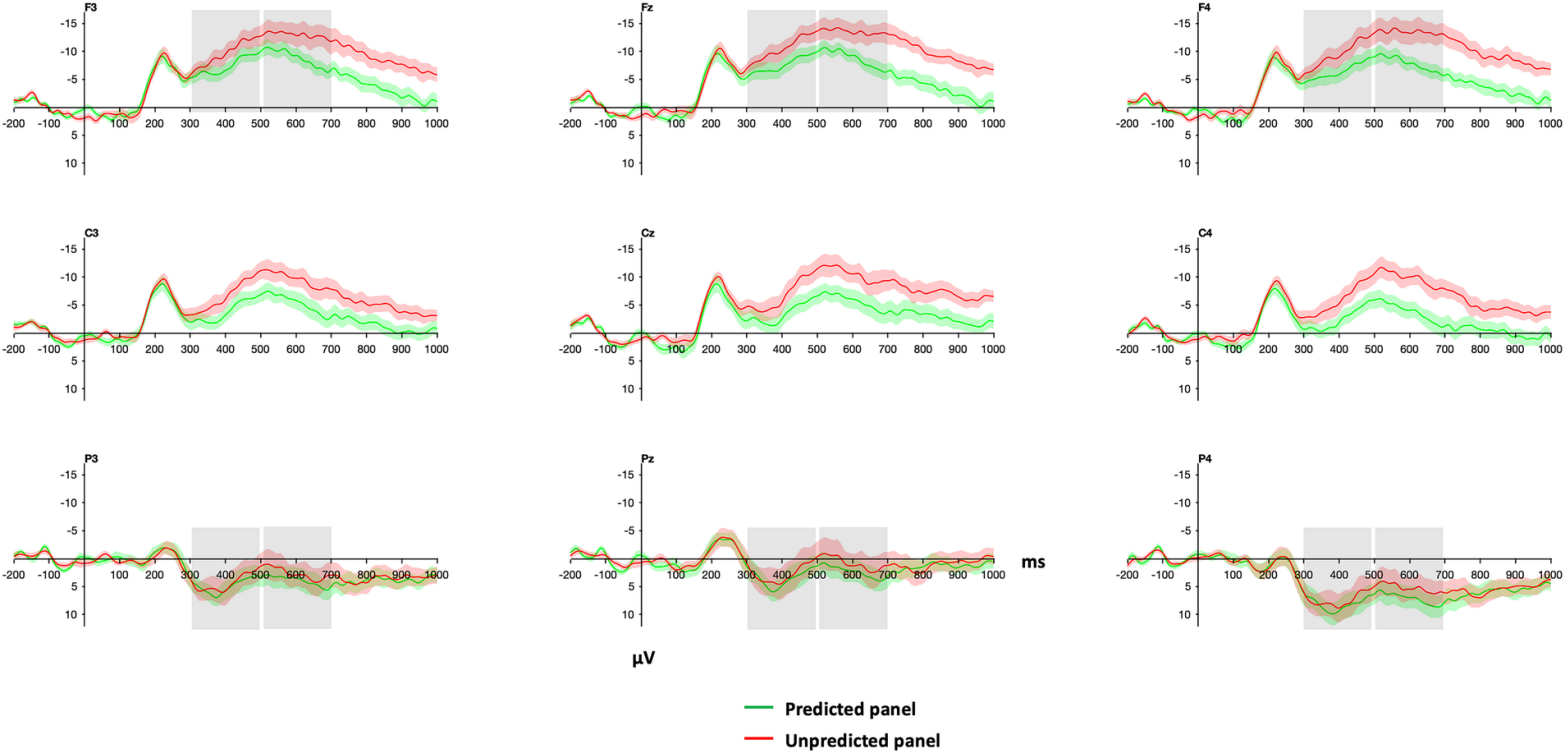
Grand average waveforms of ERPs for predicted and unpredicted panels within visual narratives. Panel onsets of critical panels are at zero ms. Grey boxes indicate the time windows and electrodes that were used in the statistical analyses (see 3.3 below). Red and green shadings indicate ±1 SE. Negative is plotted up.

Similar to the results for verbal narratives, panels elicited a more negative ERP response when unpredicted from the context than when predicted in the 300–500 ms and 500–700 ms time window (Figure 7).

### 3.3 Comparisons of N400 effects to verbal narratives and visual narratives

**Figure 8:**
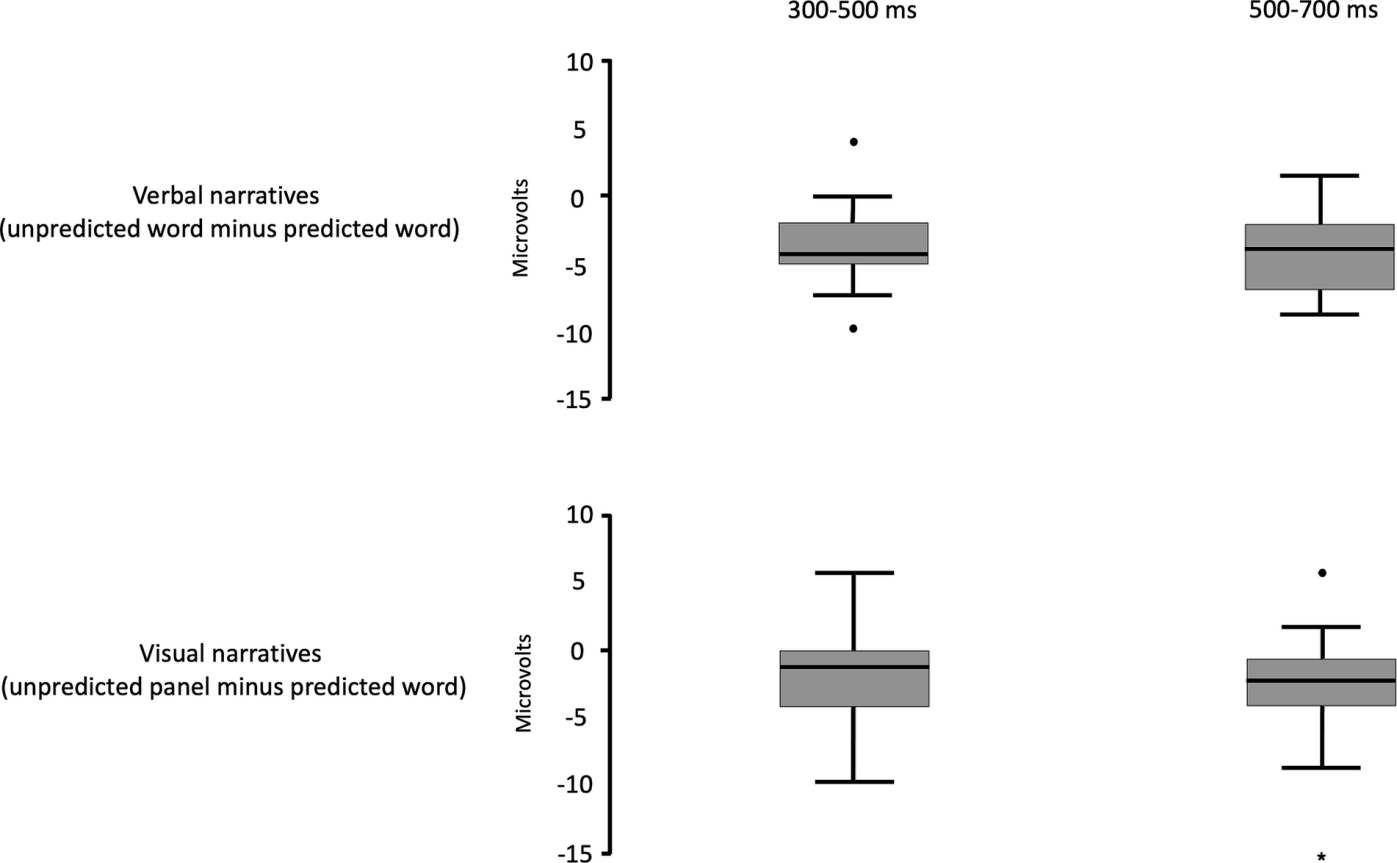
Boxplots of grand average voltage difference between unpredicted and predicted words and between unpredicted and predicted panels in the time windows 300–500 ms and 500–700 ms.

**Figure 9:**
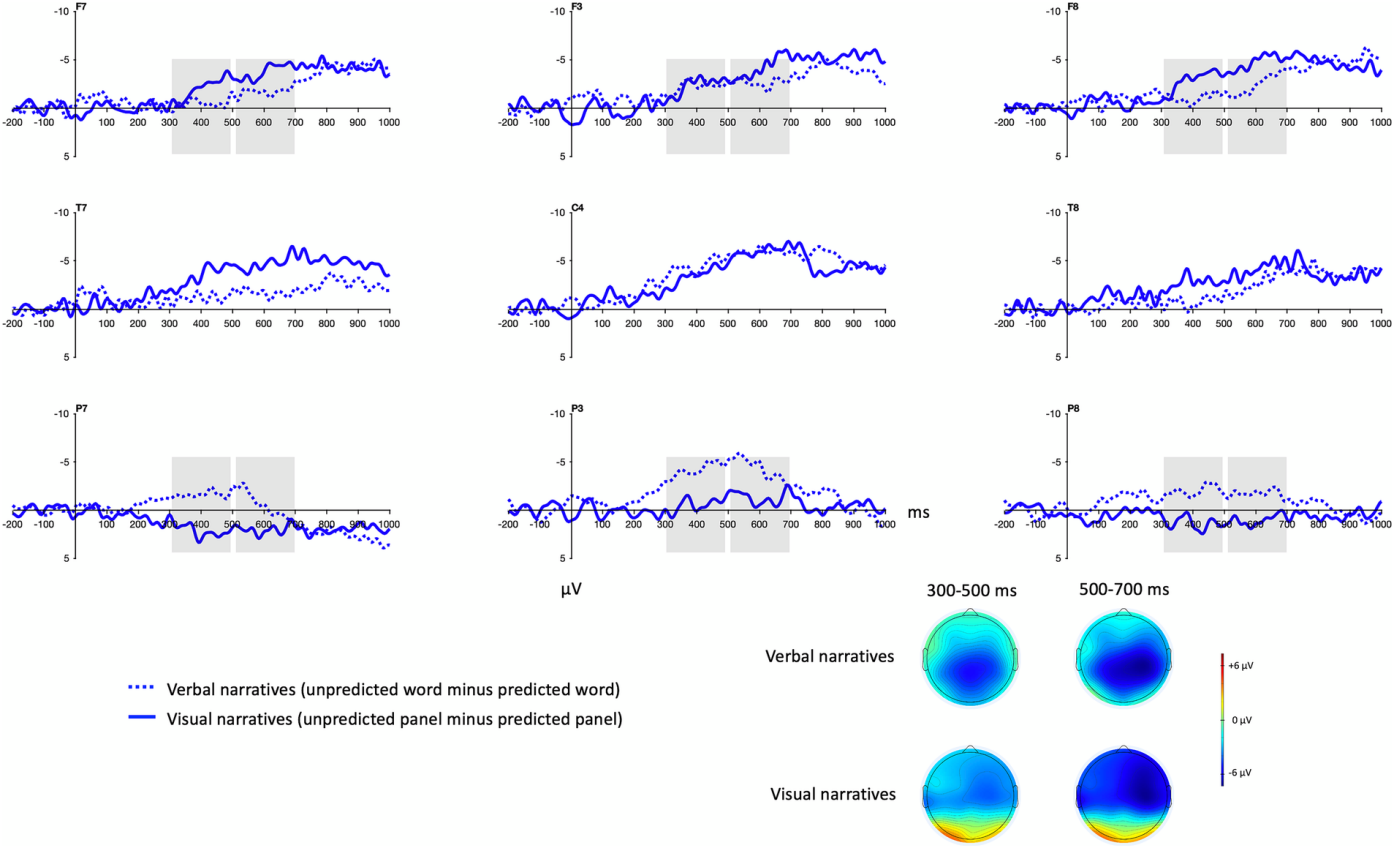
Grand average difference waves for verbal narratives and visual narratives. Dotted waveforms represent the mean voltage difference between unpredicted words and predicted words. Solid waveforms represent the mean voltage difference between unpredicted panels and predicted panels. The grey boxes indicate the time windows and the electrodes that were used in the statistical analysis. Negative is plotted up. Bottom right: Grand average topographic maps illustrate the mean voltage difference between unpredicted and predicted words and between unpredicted and predicted panels in the 300–500 ms and 500–700 ms time windows.

There was no significant difference in the mean amplitude of the N400 effect averaged over frontal and parietal sites in the 300–500 and 500–700 ms time windows between domains (Table 3 and Figure 8). The N400 effect for verbal narratives is presented in relation to the N400 effect for visual narratives with difference waves and topographic maps (Figure 9). In these figures, the N400 effect appears to be more posterior for verbal narratives than for visual narratives and, conversely, more frontal for visual narratives than for verbal narratives though statistically significant distribution differences were restricted to parietal sites (Table 3).

**Table 3:** Repeated measures ANOVA.

	df	300–500 ms			500–700 ms		
		*F*	*η_p_*^2^	*p*	*F*	*η_p_*^2^	*p*
Domain	1, 16	2.61	0.14	0.12	0.25	0.02	0.63
Domain ^*^ Distribution	1, 16	3.56	0.18	0.07	5.76	0.27	0.03
Interaction follow-up							
Frontal	1, 16	0.05	0.00	0.82	1.00	0.06	0.33
Parietal	1, 16	8.97	0.36	0.01	6.48	0.29	0.02

Dependent variable: N400 effect, i.e., mean amplitude difference in microvolts (predicted subtracted from predicted) in the 300–500 ms and 500–700 ms time-window. Independent variables: domain (verbal/pictorial) and distribution (frontal/parietal).

## 4 Discussion

We compared semantic processing of verbal narratives and visual narratives (wordless picture sequences) in school-aged children with typical language development and found the same type of processing across domains as indexed by N400 effects in both domains, in line with previous ERP studies (Kutas and Federmeier, 2011). Importantly, we directly compared the N400 effect in the verbal domain with the N400 effect in the pictorial domain and found that the distribution differed between the domains, but importantly not the magnitude of the N400 effects. This is in line with our hypothesis and has potential theoretical and practical implications. Below we discuss the results independently for each domain and the relationship between semantic processing in the two domains.

In the verbal domain, unpredicted words elicited a larger N400 than predicted words in narratives, as expected based on many previous ERP studies that have targeted semantic processing of language. However, ERP studies of children’s semantic processing in the context of verbal narratives are sparse (though see Weber-Fox et al., 2013). Although isolated sentences have been more common as stimuli in the literature, verbal narratives have arguably a higher ecological validity as everyday language use occurs in rich contexts (Hasson et al., 2018). The present study is thus an extension of these previous studies. Furthermore, this is to the best of our knowledge the first ERP study of children’s semantic processing of narratives in Swedish, which adds to our ability to generalize across languages.

Although the use of verbal narratives accompanying related animated videos increases ecological validity, it could also be considered a limitation, in that processing of visual information could be a confounding factor in the comparison between semantic processing of these verbal narratives and semantic processing of the visual narratives. However, we included N400 effects (i.e., amplitude differences for unpredicted minus predicted stimuli in each domain) in the comparisons between the domains, not N400s (i.e., amplitudes to unpredicted or predicted stimuli). Even so, it cannot be ruled out that the semantic processing of the verbal narratives was to some extent influenced by visual information in the animated videos. We determined that this was outweighed by increased ecological validity. Furthermore, the N400 effect for the verbal narratives tended to be more posterior than the N400 effect for the visual narratives, in line with the distribution of the N400 effects for auditory language and pictures in previous studies. This distribution supports our interpretation of the N400 effect for verbal narratives as an index of semantic processing in the verbal domain.

The distribution of the N400 effects for the verbal and the visual narratives cannot be connected to specific cortical regions. However, the distribution of the N400 effects can be interpreted in light of previous manipulations that affected the distribution. Pictures have repeatedly elicited more anterior N400 effects than words, which was replicated in the present study. Also, more imageable words have elicited more anterior N400 effects than less imageable words, for example *leopard* compared to *minute* (West and Holcomb, 2000; Swaab et al., 2002), while images with more abstract meanings elicit more posterior N400 effects than more imagistic images (Cohn and Foulsham, 2022). In contrast, sensory modality (visual versus auditory) has not affected the posterior distribution of the N400 effect in comparisons of written and spoken words (Hagoort and Brown, 2000). Therefore, the more anterior N400 effect for the visual narratives, compared to the verbal narratives, may be interpreted as an effect of imageability (concreteness) rather than sensory modality. This could be investigated by comparing the distribution of N400 effects for both pictures and words with varying degrees of concreteness.

The N400 effect of semantic prediction for panels within the visual narratives replicated the results in the only other ERP study of children’s semantic processing of visual narratives (Manfredi et al., 2020). However, the N400 effect in that study occurred together with a P600 effect in children with typical development but not in children with autism spectrum disorder. That biphasic N400/P600 response was surprising since an isolated N400 effect marked adults’ semantic processing of visual narratives in similar studies (West and Holcomb, 2002; Cohn et al., 2012; Coderre et al., 2018, 2020). The isolated N400 effect in the present study aligns with the results in the studies with adults and diverges from the Manfredi et al. (2020) study with children, even though they were similar in age to the children in the present study and task demands were similar (forced choice button press). However, in that study only semantic incongruencies were included, presumably making the task easier than in the current study, which included manipulations of structure. It is possible that a paradigm with manipulations of semantic congruency made it more likely that children with typical development would reanalyze the presented narratives. This occasional occurrence of a P600 effect following an N400 effect for semantic manipulations in the pictorial domain parallels the occasional occurrence of a P600 effect following an N400 effect for semantic manipulations in the verbal domain, which remains an open research question (see review by Van Petten and Luka, 2012).

The primary novelty of the present ERP study was its within-subjects design with both a verbal and a visual narrative paradigm, which allows for a detailed cross-domain comparison of children’s semantic processing in rich contexts. Indeed, there was no significant difference between the magnitude of the N400 effect for visual narratives and the magnitude of the N400 effect for verbal narratives. This result, together with behavioral studies (Zampini et al., 2017; Carlsson et al., 2020) that suggest associations between children’s verbal comprehension and visual narrative comprehension, contradict the assumption that visual narratives are easier to process than spoken languages.

The similarities of the ERP effects to verbal and visual narratives provide further support for the domain generality of semantic processing. At the same time, the scalp distribution of the N400 effects differed between the domains in that panels elicited a more frontal N400 effect than words, just as in other studies (West and Holcomb, 2002; Cohn et al., 2012; Coderre et al., 2018, 2020; Manfredi et al., 2020). Our interpretation of these results is based on previous interpretations of N400 effects (Kutas and Federmeier, 2011) – semantic processing is domain general but also domain sensitive (not domain specific), occurring in a widely distributed and dynamic representational memory system.

The present study contributes to an increased understanding of the complexity of visual narrative processing and its relation to verbal language processing in children with typical language development. This has implications for pedagogical practices. Firstly, children’s familiarity with visual narratives should be considered when these are included in pedagogical practices. Visual narratives should not be assumed to be easier to comprehend than verbal narratives. Secondly, pedagogical practices that draw on the shared features of visual and verbal narratives may be beneficial for supporting children’s language development.

## 5 Conclusion

This is the first ERP study of children’s semantic processing of verbal narratives in relation to their semantic processing of visual narratives. The results reveal similarities in semantic processing across domains as there was no significant difference in the magnitude of the N400 effect between the verbal and the pictorial domain. The only difference between the domains was the expected difference in distribution. The study contributes to an increased understanding of the complexity of visual narrative processing and its shared features with verbal language processing. Furthermore, it lays the foundation for similar studies of semantic processing across domains in children with a language disorder, where results may have implications for assessment and intervention practices.

## Data availability statement

The raw data supporting the conclusions of this article will be made available by the authors, without undue reservation.

## Ethics statement

The studies involving humans were approved by The Swedish Ethical Review Authority. The studies were conducted in accordance with the local legislation and institutional requirements. Written informed consent for participation in this study was provided by the participants’ legal guardians/next of kin.

## Author contributions

HL: Conceptualization, Data curation, Formal analysis, Investigation, Methodology, Visualization, Writing – original draft, Writing – review & editing. KH: Conceptualization, Funding acquisition, Methodology, Supervision, Writing – review & editing. EP: Conceptualization, Methodology, Writing – review & editing. NC: Conceptualization, Methodology, Writing – review & editing. AA: Conceptualization, Data curation, Formal analysis, Funding acquisition, Investigation, Methodology, Supervision, Writing – review & editing.
