# Supplementary material for: Semantic processing of verbal narratives compared to semantic processing of visual narratives: an ERP study of school-aged children
Source: Front Psychol. 2024 Jan 12;14:1253509. doi: 10.3389/fpsyg.2023.1253509 (PMC10812112; doi:10.3389/fpsyg.2023.1253509)
Supplement: Supplementary file 1 [file Data_Sheet_1.PDF]

Supplementary Material

Svensk översättning och anpassning av The Visual Language Fluency Index (VLFI)

Swedish Translation and Adaption of The Visual Language Fluency Index (VLFI)

Du ska får svara på 13 frågor för att vi ska förstå hur van du är vid tecknade serier. De första frågorna handlar om hur ofta du brukar läsa eller titta på film under en vanlig vecka. Du ska kryssa i en av rutorna för att svara på varje fråga. Vi tittar på den första frågan tillsammans. Vi hjälper dig gärna om du undrar något om de andra frågorna.

[You are to answer 13 questions to help us understand how familiar you are with comics. The first questions pertain to how often you typically read or watch films during a regular week. You should mark on of the boxes to answer each question. We will go through the first question together. We are happy to assist you with any queries about the other questions.]

1. Hur ofta brukar du läsa böcker med bara text för nöjes skull under en vanlig vecka?
[How often do you typically read books with only text for pleasure during a regular week?]

- ☐ Aldrig *[Never]*
- ☐ Nästan aldrig *[Almost never]*
- ☐ Ibland *[Sometimes]*
- ☐ Ganska ofta *[Quite often]*
- ☐ Ofta *[Often]*
- ☐ Nästan alltid *[Almost always]*
- ☐ Alltid *[Always]*

2. Hur ofta brukar du titta på film under en vanlig vecka?
[How often do you typically watch movies during a regular week?]

- ☐ Aldrig *[Never]*
- ☐ Nästan aldrig *[Almost never]*
- ☐ Ibland *[Sometimes]*
- ☐ Ganska ofta *[Quite often]*
- ☐ Ofta *[Often]*
- ☐ Nästan alltid *[Almost always]*
- ☐ Alltid *[Always]*

Supplementary Material

3. Hur ofta brukar du titta på tecknad film under en vanlig vecka?

[How often do you typically watch cartoons during a regular week?]

- ☐ Aldrig *[Never]*
- ☐ Nästan aldrig *[Almost never]*
- ☐ Ibland *[Sometimes]*
- ☐ Ganska ofta *[Quite often]*
- ☐ Ofta *[Often]*
- ☐ Nästan alltid *[Almost always]*
- ☐ Alltid *[Always]*

4. Hur ofta brukar du läsa serietidningar under en vanlig vecka?

[How often do you typically read comic books during a regular week?]

- ☐ Aldrig *[Never]*
- ☐ Nästan aldrig *[Almost never]*
- ☐ Ibland *[Sometimes]*
- ☐ Ganska ofta *[Quite often]*
- ☐ Ofta *[Often]*
- ☐ Nästan alltid *[Almost always]*
- ☐ Alltid *[Always]*

5. Hur ofta brukar du läsa seriestrippar (korta rader med serierutor) under en vanlig vecka?

[How often do you typically read comic strips (short rows of comic panels) during a regular week?]

- ☐ Aldrig *[Never]*
- ☐ Nästan aldrig *[Almost never]*
- ☐ Ibland *[Sometimes]*
- ☐ Ganska ofta *[Quite often]*
- ☐ Ofta *[Often]*
- ☐ Nästan alltid *[Almost always]*
- ☐ Alltid *[Always]*

Supplementary Material

6. Hur ofta brukar du läsa serieromaner (böcker med serier) under en vanlig vecka?
[How often do you typically read graphic novels (books with comics) during a regular week?]

- ☐ Aldrig *[Never]*
- ☐ Nästan aldrig *[Almost never]*
- ☐ Ibland *[Sometimes]*
- ☐ Ganska ofta *[Quite often]*
- ☐ Ofta *[Often]*
- ☐ Nästan alltid *[Almost always]*
- ☐ Alltid *[Always]*

7. Hur ofta brukar du läsa Manga under en vanlig vecka?
[How often do you typically read Manga during a regular week?]

- ☐ Aldrig *[Never]*
- ☐ Nästan aldrig *[Almost never]*
- ☐ Ibland *[Sometimes]*
- ☐ Ganska ofta *[Quite often]*
- ☐ Ofta *[Often]*
- ☐ Nästan alltid *[Almost always]*
- ☐ Alltid *[Always]*

8. Hur ofta brukar du själv teckna serier under en vanlig vecka?
[How often do you typically draw comics yourself during a regular week?]

- ☐ Aldrig *[Never]*
- ☐ Nästan aldrig *[Almost never]*
- ☐ Ibland *[Sometimes]*
- ☐ Ganska ofta *[Quite often]*
- ☐ Ofta *[Often]*
- ☐ Nästan alltid *[Almost always]*
- ☐ Alltid *[Always]*

Supplementary Material

9. Vilket av dessa tycker du bäst om att göra?

[Which of these do you like to do the most?]

- ☐ Läsa serietidningar *[Read comic books]*
- ☐ Läsa seriestrippar (korta rader med serierutor) *[Read comic strips (short rows of comic panels)]*
- ☐ Läsa serieromaner (böcker med serier) *[Read comic strips (books with comics)]*
- ☐ Läsa Manga *[Read Manga]*
- ☐ Teckna serier själv *[Draw comics yourself]*

10. Hur bra är du på att läsa tecknade serier (oavsett typ) i jämförelse med andra barn i din ålder?

[How good are you at reading comics (regardless of type) compared to other children of your age?]

- ☐ Bättre *[Better]*
- ☐ Lite bättre *[Slightly better]*
- ☐ Som andra *[Same as others]*
- ☐ Lite sämre *[Slightly worse]*
- ☐ Sämre *[Worse]*

11. Hur bra är du på att rita/teckna serier i jämförelse med andra barn i din ålder?

[How good are you at drawing comics compared to other children of your age?]

- ☐ Bättre *[Better]*
- ☐ Lite bättre *[Slightly better]*
- ☐ Som andra *[Same as others]*
- ☐ Lite sämre *[Slightly worse]*
- ☐ Sämre *[Worse]*

Supplementary Material

12. Hur gammal var du när du började läsa tecknade serier?

[How old were you when you started reading comics?]

- ☐ Jag har aldrig läst tecknade serier *[I have never read comics]*
- ☐ 2 år *[years]*
- ☐ 3 år *[years]*
- ☐ 4 år *[years]*
- ☐ 5 år *[years]*
- ☐ 6 år *[years]*
- ☐ 7 år *[years]*
- ☐ 8 år *[years]*
- ☐ 9 år *[years]*
- ☐ 10 år *[years]*
- ☐ 11 år *[years]*
- ☐ 13 år *[years]*

13. Hur gammal var du när du började rita tecknade serier?

[How old were you when you started drawing comics?]

- ☐ Jag har aldrig ritat tecknade serier *[I have never drawn comics]*
- ☐ 2 år *[years]*
- ☐ 3 år *[years]*
- ☐ 4 år *[years]*
- ☐ 5 år *[years]*
- ☐ 6 år *[years]*
- ☐ 7 år *[years]*
- ☐ 8 år *[years]*
- ☐ 9 år *[years]*
- ☐ 10 år *[years]*
- ☐ 11 år *[years]*
- ☐ 13 år *[years]*

Tack för hjälpen!

[Thank you for your help!]
